# MetastamiRs: Non-Coding MicroRNAs Driving Cancer Invasion and Metastasis

**DOI:** 10.3390/ijms13021347

**Published:** 2012-01-27

**Authors:** Cesar Lopez-Camarillo, Laurence A. Marchat, Elena Arechaga-Ocampo, Carlos Perez-Plasencia, Oscar del Moral-Hernandez, Elizabeth J. Castaneda-Ortiz, Sergio Rodriguez-Cuevas

**Affiliations:** 1 Genomics Sciences Program, Oncogenomics and Cancer Proteomics Laboratory, Autonomous University of Mexico City, Avenue San Lorenzo 290, 03100, Mexico; E-Mail: castajack@yahoo.com.mx; 2 Biotechnology Program, Institutional Program of Molecular Biomedicine, National School of Medicine and Homeopathy of the National Polytechnic Institute, Guillermo Massieu Helguera 239, 07320, Mexico; E-Mail: lmarchat@ipn.mx; 3 Carcinogenesis Laboratory, National Institute of Cancerology, Avenue San Fernando 22, 14080, Mexico; E-Mail: earechagao@incan.edu.mx; 4 Massive Sequencing Unit, National Institute of Cancerology, Avenue San Fernando 22, 14080, Mexico; E-Mail: carlos.pplas@campus.iztacala.unam.mx; 5 Genomics Laboratory, FES-I, UBIMED, National Autonomous University of Mexico, Avenue de los Barrios 1, 54090, Mexico; 6 Academic Unit of Biological Chemistry Sciences, Molecular Biomedicine Laboratory, Autonomous University of Guerrero, Lazaro Cárdenas S/N Col, Haciendita, Chilpancingo Guerrero, 39090, Mexico; E-Mail: odelmoral@cinvestav.mx; 7 Institute of Breast Diseases-FUCAM, Avenue Bordo 100, 04980, Mexico; E-Mail: sergiorocue@gmail.com

**Keywords:** microRNA, cancer, epithelial-mesenchymal transition, invasion, metastasis, metastamiRs

## Abstract

MicroRNAs (miRNAs) are small non-coding RNAs of ~22 nucleotides that function as negative regulators of gene expression by either inhibiting translation or inducing deadenylation-dependent degradation of target transcripts. Notably, deregulation of miRNAs expression is associated with the initiation and progression of human cancers where they act as oncogenes or tumor suppressors contributing to tumorigenesis. Abnormal miRNA expression may provide potential diagnostic and prognostic tumor biomarkers and new therapeutic targets in cancer. Recently, several miRNAs have been shown to initiate invasion and metastasis by targeting multiple proteins that are major players in these cellular events, thus they have been denominated as metastamiRs. Here, we present a review of the current knowledge of miRNAs in cancer with a special focus on metastamiRs. In addition we discuss their potential use as novel specific markers for cancer progression.

## 1. Introduction

Cancer represents a heterogeneous group of diseases characterized by uncontrolled growth of cells, high proliferation, apoptosis resistance, acquisition of invasive properties to adjacent tissues and organs, and inappropriate survival of cells, which results in tumors formation. The puzzling complexity of cancer properties was outlined as the “hallmarks of cancer” a decade ago in the seminal report of Hanahan and Weinberg [1], and it comprises six essential alterations in cell physiology that collectively dictate malignant growth: (i) self-sufficiency in growth signals; (ii) insensitivity to anti-growth signals; (iii) evasion of apoptosis; (iv) limitless replicative potential; (v) sustained angiogenesis; and (vi) tissue invasion and metastasis [1,2]. Notably, the acquired capabilities of cancer cells to invade and metastasize to other tissues and organs represent the most deadly hallmark of malignant tumors [3]. Normal cells have developed several safeguards and checkpoints to regulate the above mentioned cellular features. These cellular processes are tightly regulated by protein-encoding genes whose expression switches-on or -off during development and in the adult body. Altered versions of these genes referred to as tumor-suppressor genes and oncogenes, arise in a multistep process in cells populations that are clonally selected to cause cancer [4]. However, this view has begun to change as new molecular players in cancer have been identified, which suggests an additional level of complexity in the mechanisms leading to tumorigenesis. In the last decade, it has been reported that an abundant class of small non-coding single-stranded RNAs of ~22 nucleotides, dubbed as microRNAs (miRNA) function as negative regulators of gene expression and may have important roles in the development of cancer. An increasing number of studies are showing that the expression of miRNAs is deregulated in almost all human malignancies. Functional characterization of these aberrantly expressed miRNAs indicates that they might also function as oncogenes and tumor-suppressors, thus they have been collectively named as “oncomirs” [5]. Here we review the current knowledge of the roles of miRNAs in cancer with a special focus on miRNAs involved in metastasis, known as metastamiRs.

## 2. Mechanisms of Invasion and Metastasis

**Figure 1:**
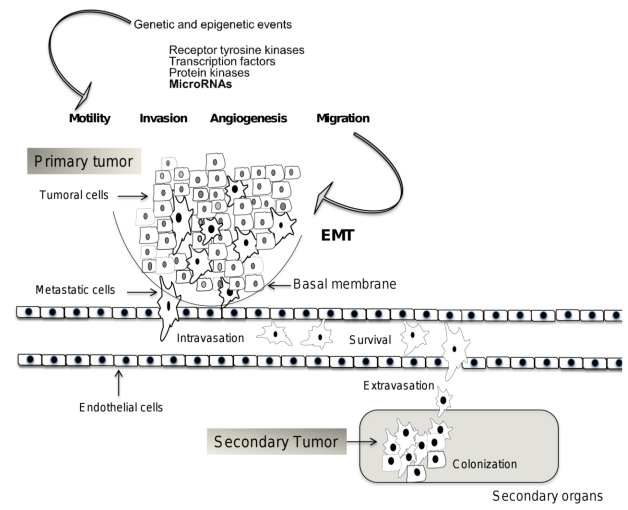
Mechanisms of invasion and metastasis. During metastatic process, tumor cells detached from primary tumor, acquire motility, degrade basal membrane, and survive in circulation within blood and lymphatic vessels. Then, circulating tumor cells are able to colonize a secondary organ, where they can survive, proliferate and form an additional tumor. Genes and molecules that participate in these mechanisms have been identified, including some miRNAs. The time between each metastatic event is variable according to primary tumor kinds, therefore molecular and epigenetic changes in tumor cells types might also be variable.

Metastasis is the result of cancer cells detaching from a primary tumor, consequently adapting to distant tissues and organs, and forming a secondary tumor [6]. The ability to metastasize is a hallmark of malignant tumors [1]. Cancer cells should successfully complete multiple sequential steps, such as spread from tumor of origin, motility, intravasation, survival in the circulation, extravasation and colonization, before they will grow and proliferate in a secondary site to form a new tumor [7,8] (Figure 1). Although it is well established that metastasis is a complex multistep event, it occurs through distinct temporal kinetics for each type of cancer, according to primary tumor origin and the ability of cells to invade specific distant tissues and organs [9]. The capability of cancer cells to metastasize depends on genetic and epigenetic events that are acquired during tumor progression [3]. Molecular and genetics modifications that conditioned cancer cell to be metastatic might depend on initial mutations, which maintain genomic instability that will generate oncogenically transformed cells [10]. Nevertheless, it remains unclear if oncogenic transformation is sufficient for metastatic competence. The long latency period of certain tumors types suggests a further evolution of malignant cells in the microenvironments of particular organs [11].

Genes and proteins participating in metastasis are related to distinct biological processes, such as interaction with the local microenvironment, migration, invasion, resistance to apoptosis, and the ability to induce angiogenesis [10,12] (Figure 1). Genes that allow cancer cell to invade the surrounding tissues attract a supportive stroma and facilitate cellular dispersion and infiltration in distant tissues, are known as metastasis initiation genes [6]. These include transcription factors, growth factors receptors, protein kinases and recently, miRNAs [13,14]. These genes participate in regulation of motility, epithelial-mesenchymal transition (EMT), adhesion and proteolysis, in order to determine tumor cell interaction with other cells and with the extracellular matrix, create a path for migration, promote angiogenesis, and both directly and indirectly trigger survival signals [15]. Cell transformation results in major phenotypic changes, including cell surface receptor expression, cytoskeletal function, growth factor and cytokine secretion, proteolytic enzyme production, and the glycosyltransferase and glycosidase repertoire expression, which affects adhesive properties [16]. Carcinoma cells interactions with the extracellular matrix are mediated by integrins and play a key role in tumor invasion and spread. Integrins trigger both signals that organize and remodel the cytoskeleton of the cell; provide polarity, and proliferation and survival control. Integrins promote invasion and proliferation and they determine whether cells migrate and proliferate in response to cytokines and growth factors [17]. Cells that become detached from the epithelium display loss of adherent junctions, which, in epithelial cells, are constituted primarily by E-cadherin. E-cadherin is replaced by N-cadherin, which plays an important role in invasion by regulating fibroblast growth factor receptor (FGFR) function [18]. This process, known as the cadherin switch, is associated with EMT. This change is referred as the conversion of epithelial cells to motile, fibroblast-like cells that express mesenchymal rather than epithelial cell markers [19].

To generate an invasive tumor, cancer cells should also enter to circulation and survive there, regardless of development of clinical metastasis. The acquisition of prometastatic function early during primary tumor formation might enable other cancer subtypes to relapse more quickly. Therefore, the typical steps of metastasis might be the same in all tumor types, including general invasive activities that are conferred by the expression of metastasis initiation genes. But metastasis to different organs might require distinct sets of infiltration and colonization functions, which are acquired over variable periods of time [6]. Accordingly, these capabilities can be provided by genes that are deregulated in primary cancer cells; these genes are known as metastatic progression genes, and they could have different functions at the primary site and in distant organ. Examples of these factors include matrix metalloproteinases (MMP), COX2 and cytokines [6]. Epithelial cell communicating with their microenvironment is regulated by E-cadherin-mediated cell-cell interaction and β1-integrin-mediated adhesion to the basement membrane (BM), which is the first barrier to invasion by carcinoma cells. MMP can degrade the various components of the BM, such as collagen IV, by proteolysis [20]. In addition to these essential roles in the early steps of tumor metastasis, MMP-2 activation promotes angiogenesis [21], one of the prerequisites for metastatic tumor growth [22]. MMP-2 degrades the fibrin matrix that surrounds newly formed blood vessels, facilitating endothelial cell penetration of tumor tissue [21]. Genes that are essential for the metastatic colonization of a certain organ become expressed only in cancer cells that have successfully achieved the previous steps of metastasis initiation and progression. Examples of these genes are parathyroid hormone-related protein (PTHRP), interleukin 11, granulocyte-macrophage colony stimulating factor (GM-CSF), interleukin 6 and TNF-α, promoting cancer cell passage through capillary walls and survival in the newly invaded tissue [23–25]. The selection for activated developmental pathways might also enhance metastatic competence to multiple organs by enforcing this plasticity and providing strong invasive and adaptation functions to cancer cells. In summary, identification of the mechanisms that promote metastatic progression without latent speciation might provide novel therapeutic targets for early intervention.

## 3. Biogenesis of microRNAs

miRNAs are small non-coding single-stranded RNAs of ~22 nucleotides in length which function at post-transcriptional level as negative regulators of gene expression, and they are found in both plants and animals (for review see [26]). miRNAs are generated as endogenous hairpin-shaped long transcripts and function as guide molecules by base pairing with the target messenger RNA (mRNA) inducing translation repression or transcripts cleavage (for review see [27]). Annotation of genomic positions of miRNAs indicates that most miRNAs genes are located in intergenic regions, but they are also found within exonic or intronic regions in either sense or antisense orientation. The miRNAs localized within introns of protein-encoding or -non-encoding genes have been denominated “mirtrons” [28]. miRNAs may be organized as individual genes or be localized together as clusters representing miRNAs families which are commonly related in sequence and function. miRNAs are transcribed by the RNA polymerase II (RNA pol II) from their own promoter or from promoter of the host gene in which they reside. RNA pol II synthesized large miRNA precursors called primary-miRNAs (pri-miRNAs) [29]. Clustered miRNAs might be transcribed from a single transcription unit as polycistronic primary-miRNA. Notably, pri-miRNAs contain both 5′-cap structure (^7^MGpppG) as well as 3′-end poly(A) tail [30]. The initial step (cropping) in pri-miRNA processing is performed in the nucleus by the enzymatic activity of an RNAse III-type protein called Drosha associated to a double stranded-RNA-binding protein DGCR8 (DiGeorge syndrome critical region gene 8; Pasha in flies) known as the microprocessor complex, that generates ~70-nucleotides precursor miRNA (pre-miRNA) products, which locally fold into stable secondary stem-loop structures [31]. The pre-miRNAs are then exported into the cytoplasm by the Ran-GTP-dependent transporter exportin 5 [32,33]. Pre-miRNA has a short stem plus a ~2-nt 3′ overhang, which is recognized by the nuclear export factor exportin 5. The second step of pre-miRNA processing (dicing) is performed by the RNAse III enzyme Dicer (Dicer 1 in flies) associated to TRBP (TAR RNA-binding protein) or PACT (also known as PRKRA), and Argonaute (AGO1-4), which cleave the miRNA precursor hairpin generating a transitory double-stranded RNA of ~22 nucleotides in length referred as miRNA:miRNA* duplex, where miRNA is known as the mature miRNA guide which remains associated to AGO, and miRNA* is denominated the passenger strand which is subsequently degraded. This duplex is then loaded into the miRNA associated RNA-induced silencing complex (RISC), which preferentially includes the mature single-stranded miRNA molecule and AGO proteins, where they act as guiding molecules to deliver the complex to target mRNA. It seems that AGO is associated to Dicer in the dicing step as well as in the RISC assembly step. The mature miRNA then hybridizes to complementary sites in the 3′ untranslated regions (3′-UTR) of mRNA targets to negatively regulate gene expression in one of two ways that depend on the degree of complementarity between the miRNA and its target. miRNAs that bind to mRNA targets with imperfect complementarity lead to translational repression and exonucleolytical mRNA decay; whereas miRNAs that bind to their mRNA targets with high complementarity induce endonucleolytical cleavage of target-mRNA [34].

**Figure 2:**
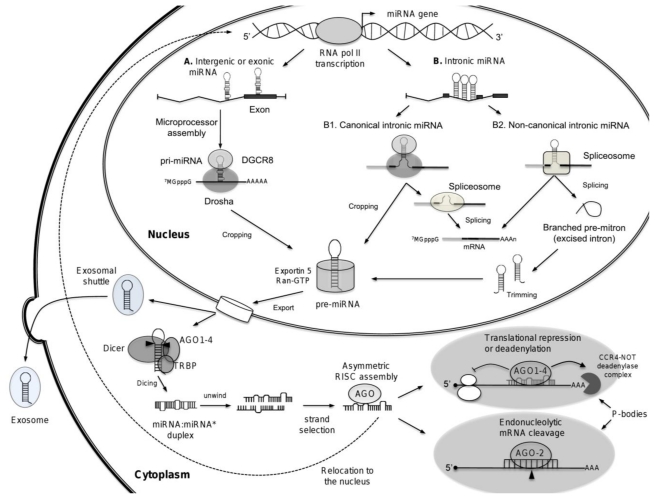
Biogenesis of microRNAs. The processing of miRNAs is mediated by Dicer and Drosha endonucleases. In the first step, the microprocessor complex (Drosha and DGCR8) mediates the nuclear processing of the primary-miRNAs (pri-miRNA) into stem-loop precursors of ~70 nucleotides (pre-miRNA). The nuclear export of the precursors is subsequently mediated by exportin-5 in a Ran-GTP dependent manner. In the second step, the pre-miRNA is cleaved in the cytoplasm by Dicer into ~22 nucleotides mature miRNA, which incorporates as single-stranded RNA into the RNA-induced silencing complex (RISC). This complex directs the miRNA to the target mRNAs, which leads either to translational repression or degradation of the target transcripts. Processing of mirtrons by alternative pathways is depicted. miRNAs-dependent repression of gene expression occurs in P-bodies which are also depicted. miRNAs are relocalized to the nucleus, where they may regulate transcription or splicing of transcripts (dotted line). Exosomal shuttle RNA is also represented as budding vesicles containing miRNAs.

Several findings suggested that miRNAs biogenesis and function are becoming more complex than previously thought. It has been proposed that canonical and non-canonical intronic miRNAs (mirtrons) follow alternative pathways for miRNA biogenesis [27]. Canonical mirtrons are processed co-transcriptionally before splicing. The splicing commitment complex is thought to tether the introns while Drosha cleaves the miRNA hairpin. The pre-miRNA enters the classical miRNA pathway, whereas the rest of the transcript undergoes precursor mRNA (pre-mRNA) splicing and produces mature mRNA for protein synthesis. In non-canonical pathway, mirtrons are produced from spliced introns as debranched introns that mimic the structural features of pre-miRNAs to enter to miRNA-processing pathway without Drosha-mediated cleavage. Because mirtrons can derive from small introns that resemble pre-miRNAs, they bypass the Drosha-processing step. Notably, some introns have tails at either the 5′ end or 3′ end, so they need to be trimmed before pre-miRNA export (Figure 2). Other reports showed the diverse functions of miRNAs. It has been reported that miRNAs may alternatively activate mRNA translation during cell cycle [34]. In addition, it has been evidenced that mature miRNAs may also be localized in nucleus, through a specific hexanucleotide (AGUGUU) sequence which acts as a transferable nuclear localization element [35]. Moreover, it has been shown that vesicles of endocytic origin known as exosomes may contain both mRNA and microRNAs, which can be delivered to another cell, and can be functional in this new location. These RNA molecules were denominated exosomal shuttle RNAs as they mediate exchange of miRNAs with other cells, which represents a novel mechanism of genetic exchange [36]. Overall, these amazing findings motivate searches for currently unidentified functions for miRNAs.

## 4. microRNAs and Cancer: oncomiRs

Recent reports have revealed that deregulation of miRNAs expression is common in human cancer and correlates with initiation and progression of tumors. Deregulation of these miRNAs, denominated as oncomiRs, is associated with genetic or epigenetic alterations, including deletion, amplification, point mutation and aberrant DNA methylation. miRNAs can act as oncogenes or tumor suppressors to inhibit the expression of cancer-related target genes and to promote or suppress tumorigenesis [5]. Those miRNAs whose expression is increased in tumors may be considered as oncogenes. These oncogene miRNAs usually promote tumor development by inhibiting tumor suppressor genes and/or genes that control cell differentiation or apoptosis [37].

In contrast, the expression of some miRNAs is decreased in cancer cells, thus they are considered as tumor suppressor genes. Tumor suppressor miRNAs usually prevent tumor development by inhibiting oncogenes and/or genes that control cell differentiation or apoptosis [37]. The identification of deregulated miRNAs in cancer and their respective targets may provide potential diagnostic and prognostic tumor biomarkers and represents new therapeutic targets for cancer therapy. In addition, large high-throughput studies in patients revealed that miRNA profiling has the potential to classify tumors and predict patient outcome with high accuracy [38]. Notably, about half of the annotated human miRNAs map within fragile regions of chromosomes, which are areas of the genome that are associated with various human cancers [39]. The initial evidence for the involvement of miRNAs in cancers came from a molecular study characterizing the 13q14 deletion in human chronic lymphocytic leukemia (CLL) [40], where they observed that two miRNAs, miR-15a and miR-16a, are located on chromosome 13q14, a region deleted in more than half of B cell chronic lymphocytic leukemia cases, which were either absent or down-regulated in the majority of CLL patients. Components of the miRNA machinery have also been implicated in tumorigenesis. Expression of Dicer has been shown to be down-regulated in lung cancer which was associated with poor prognosis [41]. Low levels of Drosha expression were significantly associated with advanced tumor stage in ovarian cancer [42]. Argonaute genes, AGO3, AGO1 and AGO4, which are clustered on chromosome 1 (1p34–35), were frequently deleted in Wilms tumors of the kidney and have also been associated with neuroectodermal tumors [43,44]. AGO1 was highly expressed in the developing lung and kidney and it was notably increased in renal tumors that lack the Wilms-tumor suppressor gene, WT1.

In summary, emerging evidence suggests that miRNAs play important roles in human cancers. Some miRNAs may be directly involved in cancer development by controlling cell differentiation and apoptosis, while others may be involved in cancers by targeting cancer oncogenes and/or tumor suppressors. Next, we will discuss the roles of miRNAs in metastasis, the more deadly hallmark in cancer.

## 5. MestastamiRs: microRNAs Regulating Metastasis

To successfully metastasize, a tumor cell must complete a complex set of processes, including invasion, survival and arrest in the circulatory system, and colonization of foreign organs [45]. Despite great advancements in knowledge of metastasis biology, the molecular mechanisms are still not completely understood. Remarkably, a regulatory role for miRNAs in metastasis has been established, thus they have been denominated metastamiRs, as they have pro- and anti-metastatic effects. The term metastamiRs was recently introduced by Welch and colleagues to refer to those regulatory miRNAs which promote or suppress various steps in migration and metastasis of cancer cells [46]. It seems that these metastasis-associated miRNAs do not influence primary tumor either in development or initiation steps of tumorigenesis, but they regulate key steps in the metastatic program and processes, such as EMT, apoptosis, and angiogenesis. Most commonly, metastamiRs promoting cell migration and invasion have been described. Because of availability of robust metastasis models, the vast majority of these metastamirs have been identified in breast and/or mammary tumor cell lines. Next, we review the identified miRNAs which have a prominent role in metastasis from a number of human cancers.

### 5.1. microRNAs in Breast Cancer Metastasis

The relevant role of miRNAs in breast cancer metastasis was established in seminal papers [47–49]. It was previously known that miR-10b was down-regulated in most breast cancers in comparison with normal mammary tissues, whereas it was highly expressed in about 50% of metastatic tumors. In 2007, Ma, *et al.* from Robert Weinberg’s group evidenced that up-regulation of miR-10b suppressed homeobox D10 (HOXD10) expression, allowing the activation of pro-metastatic gene RHOC and initiation of breast cancer invasion and metastasis [47]. Authors showed that miR-10b was 50-fold overexpressed in metastatic MDA-MB-231 cell line, in comparison with tumorigenic non-metastatic MCF7 cells. They also demonstrated that ectopic expression of miR-10b had no effect on proliferation *in vitro*, but did result in an increase in migration and invasion properties in two different human breast cell lines. In contrast, silencing of miR-10b using antisense inhibitor oligonucleotides led to a 10-fold reduction of the invasive properties from transfected cells. Importantly, overexpression of miR-10b in non-metastatic tumorigenic cell lines promoted robust invasion, and lung distant micro-metastases *in vivo*. In addition, miR-10b-overexpressing tumors exhibited high levels of cell proliferation and they were highly vascularized. Then, Ma and coworkers showed that TWIST1, a metastasis promoting transcription factor [50] specifically binds to the putative promoter of *mir-10b* gene activating its expression. Moreover, this induced the translation inhibition of homeobox HOXD10 transcription, resulting in an increased expression of the prometastatic gene RHOC (Ras homolog gene family member C). HOXD10 is a type I homeobox transcription factor that maintains a differentiated phenotype in epithelial cells and is essential for morphogenesis and differentiation control [51]. Notably, HOXD10 expression was found lost in breast tumors showing increasing degrees of malignancy [52]. RHOC is a signaling GTPase-protein involved in metastasis that is repressed by HOXD10. Ma and coworkers showed that reduction in RHOC expression by small interfering RNA caused repression of miR-10b induced cell migration and invasion, which indicates that RHOC is a downstream effector of miR-10b. Besides, it was recently reported that systemic antagomirs-based therapeutic silencing of miR-10b in tumor-bearing mice suppressed breast cancer metastasis. Silencing of miR-10b significantly decreases miR-10b levels and increases the levels of its target HOXD10 [53].

The second hallmark report [48] established that miR-373 and miR-520c can also promote tumor invasion and metastasis by regulating the cell-surface glycoprotein encoding gene CD44 (cell surface receptor for hyaluronan). In breast cancer cells, the metastatic cell state is strongly correlated to EMT and the CD44+/CD24− stem cell phenotype. Cell lines with high CD44+/CD24− cell numbers are basal/mesenchymal or myoepithelial types and are more invasive than other cell lines [54]. In order to identify new potential metastasis-promoting miRNAs, Huang, *et al*. from Agami’s group set up a genetic screen using the non-metastatic MCF7 cell line, and found that miR-373 and miR-520c stimulated cell migration and invasion *in vitro* and *in vivo*. Similarly to miR-10b, miR-373 and miR-520c did not affect cell proliferation. Previously, the same group identified miR-373 as a potential oncogene in testicular germ-cell tumors where it suppressed the oncogene-induced p53 pathway, which cooperates with oncogenic RAS to promote cellular transformation [55]. Interestingly, Huang and coworkers found that miR-373 and miR-520c “seed” sequences were similar and both target CD44 messenger RNA. Moreover, enhanced expression of a CD44 gene that was unresponsive to miR-373/miR-520c inhibited the migratory activity of MCF7 cells overexpressing miR-373 and miR-520c. Finally, they analyzed the miR-373 expression in breast cancer clinical specimens and found a significant up-regulation of miR-373 in 11 matched normal/tumor samples, which inversely correlated with CD44 expression, in particular in tumors exhibiting lymph node metastasis.

The third outstanding study reports the identification of new miRNAs involved in breast cancer metastasis [49]. The team led by Joan Massague found that miR-335, miR-126, and miR-206 are metastasis-suppressors. Authors performed array-based miRNA profiling in MDA-MB-231 breast cancer cell derivatives highly metastatic to bone and lung, and found a signature of six genes (miR-335, miR-126, miR-206, miR-122a, miR-199a*, and miR-489) whose expression was highly decreased in metastatic cells. Restoring the expression of miR-335, miR-126 or miR-206 in LM2 cells decreased the lung colonizing activity of these cells by more than fivefold. Interestingly, miR-126 restoration reduced overall tumor growth and proliferation, whereas miR-335 inhibited metastatic cell invasion. In addition, low expression of miR-335 or miR-126 in primary tumors from patients was associated with poor distal metastasis-free survival. To obtain more insights about the miR-335 loss consequences, authors transfected MDA-MB-231 cells with an anti-miRNA antagomir targeting either miR-335, miR-199a* or a control sequence, and found that inhibition of miR-335 enhanced the lung-colonizing ability of cells. Then, they profiled LM2 cells overexpressing miR-335 and identified 756 genes whose expression was decreased when compared with control LM2 cells, including genes previously implicated in extracellular matrix and cytoskeleton control (type 1 collagen COL1A1) and signal transduction (receptor-type tyrosine protein phosphatase PTPRN2, c-Mer tyrosine kinase (MERTK) 21 and phospholipase PLCB1), as well as in cell migration, such as the tenascin C (TNC), an extracellular matrix glycoprotein of stem cells niches [56] and the SRY-box containing transcription factor SOX4 [57]. These experimental evidences indicated that miR-335 suppresses metastasis and migration by targeting the progenitor cell transcription factor SOX4 and TNC messenger RNAs. Consequently, loss of miR-335 leads to the activation of SRY-box containing SOX4 and TNC, which are responsible for the acquisition of metastatic properties. Notably, knockdown of SOX4 and TNC using RNA interference diminished *in vitro* invasive ability and *in vivo* metastatic potential, evidencing that both genes are key effectors of metastasis [49]. Recently, Tavazoie’s group reported that miR-126 regulates endothelial cell recruitment to metastatic breast cancer cells, *in vitro* and *in vivo*. They evidenced that miR-126 suppresses metastatic endothelial recruitment, metastatic angiogenesis and metastatic colonization through coordinate targeting of insulin-like growth factor binding protein 2 (IGFBP2), PITPNC1 and *c*-Mer tyrosine kinase (MERTK) receptor genes [58].

In addition, others miRNAs with prominent roles in breast cancer metastasis have been reported. miR-146a and b inhibited invasion and migration of breast cancer cells by down-regulating NFκB through IRAK1 and TRAF6 targeting [59]. These studies were extended *in vivo* by showing both miR-146a and b suppressed metastasis that may involve targeting of EGF receptor or ROCK1 [60] both of which are involved in promoting invasion and metastasis. Hurst and coworkers showed that breast cancer metastasis suppressor 1 (BRMS1), a protein that regulates expression of multiple genes leading to suppression of metastasis, significantly up-regulates miR-146a and miR-146b in metastatic breast cancer cells. Transduction of miR-146a or miR-146b into MDA-MB-231 down-regulated expression of epidermal growth factor receptor, inhibited invasion and migration *in vitro*, and suppressed experimental lung metastasis [61]. In addition, it was reported that miR-31 inhibited multiple steps of metastasis including invasion, anoikis, and colonization leading to almost complete reduction in lung metastasis [62]. Clinically, miR-31 levels were lower in breast cancer patients with metastasis. In another study, it was reported that suppression of miR-21 in metastatic MDA-MB-231 breast cancer cells significantly reduced invasion and lung metastasis, by targeting programmed cell death 4 (PDCD4) and maspin, both of which have been implicated in invasion and metastasis. Li and coworkers reported that down-regulation of miR-193b contributes to enhance urokinase-type plasminogen activator expression and tumor progression and invasion in human breast cancer [63]. By profiling of primary breast tumors, tumor associated normal tissue and breast cancer cell lines using RQ-PCR, as well as functional assays, it was demonstrated that miR-183 targets ezrin and may play a central role in the regulation of migration and metastasis in breast cancer [64]. Sachdeva and Mo reported that miR-145 suppresses cell invasion and metastasis by directly targeting the metastatic gene *mucin 1*. Moreover, they showed that suppression of MUC1 by miR-145 causes a reduction of β-catenin as well as the oncogenic cadherin 11 [65]. Yu and colleagues demonstrated a unique mechanism of how the altered microRNA17/20 expression regulates cellular secretion and tumor microenvironment to control migration and invasion of neighboring cells in breast cancer. They showed that miRNA17/20 directly represses IL-8 by targeting its 3′ UTR, and inhibits cytokeratin 8 via the cell cycle control protein cyclin D1 [66]. In addition, miR-9, which is up-regulated in breast cancer cells, directly targets CDH1, the E-cadherin-encoding messenger RNA, leading to increased cell motility and invasiveness [67]. Remarkably, it was reported that overexpression of miR-200, which promotes a mesenchymal to epithelial cell transition by inhibiting Zeb2 expression, unexpectedly enhances macroscopic metastases in mouse breast cancer cell lines. These findings were surprising since the miR-200 family was previously shown to promote epithelial characteristics by inhibiting the transcriptional repressor Zeb2 and thereby enhancing E-cadherin expression [68]. In another study, Vetter and coworkers showed that miR-661 expression in MCF7 breast cancer cells conditionally overexpressing the EMT master regulator SNAI1 contributes to breast cancer cell invasion by targeting cell-cell adhesion Nectin-1 and the lipid transferase StarD10 messengers [69]. Yong Li and coworkers found that miR-196 inhibited the expression of HOXC8 transcription factor, which suppressed cell migration and metastasis. Importantly, unlike other metastasis-associated miRNAs that have been described, the expression of miR-196 was not correlated with breast cancer cell migration or the metastatic status of clinical breast tumor specimens. Instead, they detected a correlation between the ratio of miR-196 to HOXC8 expression and the migratory behavior of breast cancer cell lines, as well as the metastatic status of clinical samples [70].

### 5.2. MetastamiRs in Lung Cancer

Lung cancer is the leading cause of cancer-related mortality in men and women worldwide. The most common lung cancer subtype is non-small cell lung cancer (NSCLC). 75% of the lung cancer patients present local and/or distant metastases with median survival by 3 to 5 months [71]. Several studies have documented the implications of miRNAs in nearly every carcinogenesis process of lung cancer. Overexpression or suppression of specific miRNAs can regulate the biological alterations during lung carcinogenesis, such as development and growth tumor, resistance to anti-cancer drug, apoptosis, as well as invasion and metastasis. Investigations of miRNAs targets and pathways that they regulate, which include known tumor suppressors and oncogenes, can explain their effects to either promote or inhibit metastatic potential in lung cancer [72]. EGFR is one of the most common mutated genes in NSCLC, which has been used as a clinical predictor of therapeutic response. EGFR dependent signaling pathways are responsible for regulation of many biological features of NSCLC, including metastases [73]. MiR-125a-5p was reported to inhibit migration and invasion of lung cancer cells by regulating the expression of several downstream genes of EGFR signaling [74]. Furthermore, miRNA profiling has linked miR-125a-5p expression to metastatic tumor features and lung cancer patient outcome, thus having a potential diagnostic and prognostic impact [75]. Down-regulation of miR-125a-3p and miR-125a-5p in NSCLC could predict a more aggressive clinical course by promoting tumor invasion and lymph node metastasis [76].

miR-183 was identified as a negative regulator of lung cancer metastasis from screening with a miRNA array. Expression level of miR-183 was demonstrated to reversely correlate with the metastatic potential of lung cancer cells by targeting VIL2-coding protein ezrin and regulating the expression of other genes involved in migration and invasion. Ezrin, which was confirmed by luciferase reporter gene assay, has a role in controlling the actin cytoskeleton, cell adhesion and motility [77]. Sarver and collaborators found that the expression levels of miR-183, as well as miR-96 and miR-182, were higher in A549 and 95D metastatic lung cancer cells than in H1299 and 95C non-metastatic cells, respectively, suggesting that the miR-183 family might participate in tumor metastasis [78]. Moreover, members of the miR-183 family were highly expressed in lung cancer primary tissues and sera. Overexpression of miR-182 and miR-183 in NSCLC tumors was strongly associated with metastasis. Notably, miR-183 was associated to lymph node metastasis, invasion of the lung membrane, and advanced clinical stage of NSCLC, while overexpression of miR-182 was positively related to invasion of the lung membrane and tumor size >3 cm in NSCLC [79].

miR-126 that was previously described as a potential metastasis suppressor miRNA in human breast cancer [49] was also found down-regulated in lung cancer [80]. Experimentally, overexpression of miR-126 in lung cancer cell line resulted in a decrease of Crk protein that belongs to the family of adaptor proteins involved in intracellular signal pathways altering cell adhesion, proliferation, and migration. Elevated levels of Crk expression have been implicated in lung cancer and are associated with increased tumor invasiveness, which provided clinical relevance to these findings. miR-126 can decrease adhesion, migratory and invasive capacity of lung cancer cell lines through posttranscriptional silencing Crk [81].

As an early step during metastasis, EMT also seems to be tightly regulated by miRNAs, mainly the miR-200 family [82]. miR-200 inhibits lung adenocarcinoma cell invasion and metastasis by targeting Flt1/VEGFR1 in a mouse model [83], while miR-221 and 222 enhance cellular migration through activation of the AKT pathway [84]. miRNAs expression in tumoral tissues, serum and blood in relation with their metastatic potential might have a clinical impact. The serum level of *SERPINB5*, *TPM1*, *RECK*, *TIMP3* miRNAs could be identified as an indicator for metastatic NSCLCs and may be applied clinically in combination with other lung cancer-specific miRNAs, such as miR-21, miR-210, and miR-486-5p in the future [85]. Overexpression of miR-21 further stimulates invasion, intravasation and metastasis through targeting of *SERPINB5*, *TPM1*, *RECK*, and *TIMP3* genes [86,87].

Very recently, Wang and coworkers analyzed miRNA expression profiles in NSCLC and identified 40 differentially expressed miRNAs. miR-451 was the most down-regulated in NSCLC tissues [88]. Moreover, low expression level of *miR-451* was found to be significantly associated with NSCLC tumor differentiation, pathological stage, lymph node metastasis, and shorter overall survival of patients. Data also indicated that miR-451 regulates survival of NSCLC cells partially through the down-regulation of RAB14, which suggested that targeting miR-451/RAB14 interaction might serve as a novel therapeutic application to treat NSCLC patients [88].

Epigenetic regulation is a mechanism to inhibit the expression of miRNAs, Lujambio, *et al*. reported that methylation of miR-9-3 was associated with lymph node metastasis. Among the miRNAs that were reactivated upon drug treatment based on demethylation, miR-148a, miR-34b/c, and miR-9 were found to undergo specific hypermethylation-associated silencing in cancer cells compared with normal tissues [89]. The reintroduction of miR-148a and miR-34b/c in cancer cells with epigenetic inactivation inhibited cell motility, reduced tumor growth, and inhibited metastasis formation in xenograft models, with an associated down-regulation of the miRNA oncogenic target genes, such as C-MYC, E2F3, CDK6, and TGIF2. Particularly, the involvement of miR-148a, miR-34b/c, and miR-9 hyper-methylation in metastasis formation was also suggested in human primary malignancies because it was significantly associated with the appearance of lymph node metastasis. These findings indicated that DNA methylation-associated silencing of tumor suppressor miRNAs contributes to the development of human cancer metastasis.

Identification of a metastatic miRNA signature offers an exciting new insight into the molecular mechanisms underlying cancer metastasis and it may represent a novel diagnostic tool in the characterization of metastatic cancer gene targets. Baffa and collaborators identified a global miRNA expression signature that can distinguish primary colon, bladder, breast, and lung cancers from their corresponding metastasis to lymph nodes, confirming a direct involvement of miRNAs in cancer metastasis [90]. Authors performed a miRNA microarray analysis and they identified a metastatic cancer miRNA signature comprising 15 overexpressed and 17 repressed miRNAs. Up-regulated miRNAs in NSCLC included miR-142-5p, miR-148b, miR-148a, miR-369-3p, miR-215, miR-152 and miR-155, whereas down-regulated miRNAs were miR-373 and miR-138-I. Some of these miRNAs have a well-characterized association with cancer progression, e.g., miR-10b, miR-21, miR-30a, miR-30e, miR-125b, miR-141, miR-200b, miR-200c, and miR-205 [90].

Almost 25% of patients with NSCLC will have brain metastasis. Analysis of expression profiles of miRNAs obtained from lung tumors samples that are able to develop brain metastasis revealed that miR-328 was associated with higher risk for developing brain metastasis. *In vitro* studies using lung cancer cells showed that miR-328 enhances cell migration, which confirmed the relevance of miRNAs in NSCLC metastatic process [91].

### 5.3. MetastamiRs in Prostate Cancer

Prostate cancer is the second more lethal cancer type in men in America [71]. Patients with prostate tumors often present multifocal localized disease that can have a long indolent period of 10–15 years before it progresses to metastatic disease. The principal metastasis site for prostate tumors is bone; once it has progressed to metastasis, the disease is currently incurable, since metastatic cells are highly resistant to conventional therapies. This suggested that prostate tumor cells have a latency period more extensive than other cancer types for which the latency period between primary and metastatic disease is relatively short. The involvement of miRNAs in human prostate cancer has been well documented and some aberrantly expressed miRNAs have been discovered in cell lines, animal models and clinical tissues from prostate tumors. These miRNAs may play critical roles in the development and progression of prostate cancer, including metastatic events [92]. MiR-21 is one of the more extensively studied miRNAs in many tumors types including prostate cancer, producing a more marked effect on tumor metastasis [93]. The level of miR-21 expression significantly correlates with advanced clinical stage, metastasis and poor prognosis in these tumors. Studies using prostate cancer cells have shown that miR-21 targets myristoylated alanine rich protein kinase c substrate (MARCKS), which is involved in cellular processes, such as cell adhesion and cell motility through regulation of the actin cytoskeleton. Notably, cell proliferation was not affected, which suggested that molecules that are regulated by miR-21 might be important to metastatic process in prostate cells [94]. Recently, Watahiki, *et al.* obtained miRNA profiles through massively parallel DNA sequencing from a transplantable metastatic compared with non-metastatic prostate cancer xenograft line, both derived via subrenal capsule grafting, and from one patient’s primary cancer tissue. Differentially expressed known and novel miRNAs were detected that might have specific roles in the metastasis of prostate cancer. Predicted target genes of these miRNAs were related to cancer and metastasis, confirming their potential role in these events in prostate cancer cells [95]. Some studies suggested that miR-205 specifically suppresses the expression of ErbB3 and VEGF-A, which are highly related to metastasis and invasion. Apparently, miR-205 interacts with its putative binding site at the 3′-UTR of both genes [96]. Gandellini and coworkers have suggested that miR-205 is overexpressed in normal prostate tissue and RWPE-1 cells, whereas it is almost undetectable in both androgen-dependent and androgen-independent prostate cancer cells. Moreover, overexpression of miR-205 in prostate cancer cells promotes up-regulation of E-cadherin and reduction of cell locomotion and invasion, suggesting a relation with EMT. Target prediction analysis indicates that miR-205 could regulate N-chimaerin, ErbB3, E2F1, E2F5, ZEB2, and protein kinase-C epsilon expression [97].

Another report demonstrated that miR-101 plays a major role in EMT through enhancement of a histone methyl transferase Zeste homolog (EZH2) whose expression was found elevated in a subset of aggressive, clinically localized prostate cancers and almost all metastatic prostate cancers. miR-101 locus is lost in metastatic prostate cancer, which leads to up-regulation of EZH2, indicating that miRNA-101 acts as a EZH2 repressor [98]. Up-regulation of EZH2 through miR-101 induces the concomitant deregulation of epigenetic pathways and results in cancer progression [99].

Recently, Peng and colleagues reported that the expression of five miRNAs, namely miRs-508-5p, -145, -143, -33a and -100, was significantly decreased in bone metastasis when compared with primary tumor prostate. Two miRNAs, miRs-143 and -145, were up-regulated, and they were able to repress migration and invasion *in vitro*, tumor development and bone invasion *in vivo,* as well as EMT of PC-3 derived from metastatic cells. Since the principal problem arising from prostate cancer is its propensity to metastasize to bone, these findings could be important for the understanding of metastasis organ-specific in prostate cancer [100].

### 5.4. MetastamiRs in Colorectal Cancer

There are currently about 100 miRNAs implicated in colorectal cancer (CRC) alone [101]. The most up-regulated miRNAs are: miR-21, miR 17-92 cluster, miR-135a/b, miR-471 and miR-675, whereas miR-143, miR-14, let-7 and miR-101 showed a decreased expression in CRC. All of them affect different target genes and have been experimentally validated. The main targets of these miRNAs include transcription factors like c-MYC, STAT, OCT4, SOX, E2F1, ZEB1, ZEB2 and NFIB, some kinases, such as ERK and YES1, and other proteins involved in matrix metalloproteinasas regulation like RECK and TIMP3 that serve as metastasis suppressors [102]. The extracellular matrix remodeling is one of the necessary conditions for tumor growth, invasiveness and metastasis, and it has been reported that up-regulation of miR-21 in CRC cells increases their migratory and invasive abilities. The mechanism is not well elucidated but, it could be through regulation of RECK and TIMP3 genes like in glioblastoma model. Then miR-21 could be implicated in extracellular matrix breakdown [103,104].

Using a mouse model of colon cancer, Dews, *et al*. demonstrated that the angiogenic activity of c-MYC is due at least in part to downstream activation of the miR-17-92 cluster. The anti-angiogenic factors, thrombospondin-1 (TSP1) and connective tissue growth factor (CTGF), are negatively regulated by these miRNAs, which are potently induced by c-MYC in this model. Robust vascularization of tumors can be induced by expression of either c-MYC or the miR-17-92 cluster [105].

### 5.5. MetastamiRs in Gastric Cancer

Human gastric cancer (GC) is an aggressive and lethal malignancy and is one of the most frequent around the world. Recent studies have shown an association of miRNAs expression and different malignancies but their prognostic role in gastric cancer is poorly understood. The first report about the association between miRNAs and gastric cancer was published in 2009 by Guo and collaborators [106]. These authors showed that miRNAs 20-b, miR-20a, miR-17, miR-21, miR-106a, miR-18a, mir-21, miR106b, mir-18b, miR-421, miR-340, miR-19a and miR-658 were highly expressed in GC. Among them, miR-340*, miR-421 and miR-658 were first found highly expressed in cancer cells. In addition, the expression of some target genes, such as *Rb* and *PTEN*, was predicted in GC tissues. More recently, Brenner and collaborators analyzed and compared miRNAs profiles between primary tumor of patients with recurrent and non-recurrent GC and reported that three miRNAs (miR-451, miR-199a-3p and miR195) were differentially expressed in tumors from patients with good prognosis in comparison with patients with bad prognosis [107]. Of these, miR-451 had the strongest prognostic impact. On the other hand, the overexpression of miR-27 in GC tissues has been related to increased levels of epithelial-mesenchymal transition-associated genes *ZEB1*, *ZEB2*, slug and vimentin, as well as to decreased E-cadherin levels. As we mentioned above, epithelial-mesenchymal transition (EMT) is a key step toward cancer metastasis. These data indicated that miR-27 promotes gastric cancer metastasis, activating MET and Wnt/b-catenin pathways, suggesting a potential application of miR-27 for gastric cancer therapy [108].

Liang and coworkers demonstrated that overexpression of let-7f in gastric cancer could inhibit invasion and migration of GC cells through direct targeting of the tumor metastasis-associated gene *MYH9* [109]. These authors used qRT-PCR experiments to evidence the low expression levels of let-7f in metastatic gastric cancer tissues and cell lines that are potentially highly metastatic. Using xenograft models of GC in nude mice, they confirmed that let-7f could inhibit gastric cancer metastasis *in vivo* after transfection by the lentivirus pGCsil-GFP- let-7f. miR-148a functions as a tumor metastasis suppressor in GC, and its down-regulation contributes to GC lymph-node metastasis and progression. The authors examined miR-148a levels in 90 gastric cancer samples by qRT-PCR and showed clinicopathological significance with miR-148a expression. They also found that GC cells transfected with miRNA-148a had reduced migration and invasion capacities *in vitro* and metastasis ability *in vivo*. In addition, overexpression of miR-148a in GC cells reduces mRNA and protein levels of *ROCK1*, whereas miR-148a silencing significantly increased *ROCK1* expression [110].

Deregulation of others miRNAs, such as miR-622, miR-107, miR-221, and miR-222, has been described in GC. MiR-622 expression was down-regulated in gastric cancer whereas ectopic expression of miR-622 promoted invasion, tumorigenesis and metastasis of GC cells both *in vitro* and *in vivo*. A luciferase reporter assay showed the effect of miR-622 on inhibitor of growth family, member 1 (*ING1*) expression [111]. The expression of miR-107 was found inversely correlated with *CDK6* expression in GC cell lines. The authors argued that miR-107 could significantly suppress *CDK6* 3′-UTR luciferase reporter activity. Ectopic expression of miR-107 reduced both mRNA and protein expression levels of *CDK6*. Moreover, it inhibited proliferation, induced G1 cell cycle arrest, and blocked invasion of the gastric cancer cells [112]. *DICER1* and *PTEN* genes have been identified as target genes for miR-107 and miR-222, respectively [113,114]. miR-101 was found down-regulated in gastric cancer tissues, and its ectopic expression significantly inhibited cellular proliferation, migration, and invasion of GC cells but its gene target has not been identified yet [115].

Finally, an interesting study published by Wu and colleagues reported that the expression of thirty-eight miRNAs in gastric cancer tissues was significantly different from that observed in paired normal tissues, using stem-loop real-time reverse transcription-polymerase chain reaction [116]. Among them, 31 miRNAs were found to be overexpressed in cancer tissues and one miRNA was ≥1.5 fold down-regulated. Particularly, the authors concluded that miR-212 and miR-195 could be independent biomarkers to predict gastric cancer metastasis to lymph node.

### 5.6. MetastamiRs in Head and Neck Cancer

Head and neck cancer includes biologically similar tumors of the nasal cavities, paranasal sinuses, oral cavity (inner lip, tongue, floor of mouth, gingivae, and hard palate), nasopharynx, oropharynx, hypopharynx, and larynx. Almost all these tumors are squamous cell carcinomas (SCC) arising from mucosal surfaces. Head and neck squamous cell carcinoma (HNSCC) is one of the most common cancers worldwide, with about 500,000 new cases each year [71]. HNSCC usually are asymptomatic until they spread to the lymph nodes of the neck, which often represents the first diagnosis element at a late stage of the disease that is characterized by invasion and metastasis events in the head and neck area. Prognosis remains poor for most HNSCC patients, with only five years survival.

By comparison of miRNA expression profiles from six paired HNSCC cell lines (UM1/UM2, 1386Tu/1386Ln and 686Tu/686Ln) with different metastatic potential, Liu and colleagues [117] evidenced a set of differentially expressed miRNAs. Some of them have been previously implicated in tumorigenesis and metastasis, while the role of others in tumorigenesis is not clear. Particularly, lower miR-138 levels were consistently observed in all highly invasive cell lines. Down-regulation of miR-138 has been previously observed in tongue squamous cell carcinoma (TSCC) [118] and thyroid carcinoma [119]. Ectopic transfection of miR-138 mimic suppressed cell invasion and led to cell cycle arrest and apoptosis. In contrast, knockdown of miR-138 enhanced cell invasion and suppressed apoptosis. Two putative genes for miR-138 precursors have been located on chromosome 3p21.33 and 16q13, respectively, in human genome. Notably, loss of heterozygosity at both chromosome loci is a frequent event that has been associated to HNSCC progression and metastasis [120–122], which could be due to reduced levels of miR-138.

Using the same TSCC UM1/UM2 cell lines and microarray assays, another group reported the down-regulation of 23 miRNAs and the up-regulation of 22 miRNAs in highly metastatic UM1 cells [123]. Down-regulated miRNAs included miR-222 that has been previously suggested to play a role in tumorigenesis by targeting cell cycle inhibitor p27, which leads to deregulation of cell cycle control in several cancer types [124,125]. Transfection of UM1 cells with the hsa-miR-222 mimic did not modify apoptosis or cell cycle, but led to a significant decrease in cell invasion (*p* < 0.05). In addition, a targeting site for hsa-miR-222 was identified in the 3′-UTR of the matrix metalloproteinase 1 (MMP1) and manganese superoxide dismutase 2 (SOD2) mRNA sequences by *in silico* analysis and confirmed by luciferase reporter gene assays. Ectopic transfection of hsa-miR-222 induced a decrease in MMP1 and SOD2 levels in UM1 cells. Furthermore, MMP1 and SOD2 independent knockdown by siRNA evidenced that the reduced MMP1 and SOD2 levels are associated with reduced cell invasion, which suggested that hsa-miR-222 regulates TSCC invasion by targeting MMP1 and SOD2 mRNA. In addition, SOD2 knockdown led to the down-regulation of *MMP1* expression, in agreement with previous observations that SOD2-dependent production of H_2_O_2_ regulates expression of MMP family members (including MMP1) and contributes to cancer cell metastasis [126–128].

The study of 31 laser-microdissected nasopharyngeal carcinomas (NPC) and 10 healthy surrounding nasopharyngeal epithelial cells through highly sensitive microarray-based procedures evidenced the differential expression of miR-29c (>5-fold) [129]. Computational approaches and analysis of mRNA expression profiling data of the same specimens, allowed the identification of putative miR-29c target genes, which are involved in extracellular matrix synthesis or its functions, including seven collagens and laminin γ1, that are associated with increased invasiveness in culture and increased metastasis in animal models and multiple human solid tumors as well as fibrillin and secreted protein, acidic, cysteine-rich (SPARC). Interestingly, the >5-fold down-regulation of mir-29c level correlated with 2- to 6-fold increase of target mRNAs in NPC tumors, which could facilitate rapid matrix generation and renewal during tumor growth and the acquisition of tumor motility. In cultured cells, introduction of miR-29c led to a reduced transcription of these genes. Moreover, luciferase reporter gene assays confirmed that miR-29c interacts with their 3′ UTR [130–135].

miR-10b has been involved in breast cancer invasion and metastasis [39]. Interestingly, its expression can be induced directly by the transcription factor Twist, a key molecule for nasopharyngeal carcinoma (NPC) metastasis [136] and the expression of Twist can be induced by Latent membrane protein-1 (LMP1) encoded by Epstein-Barr virus (EBV) that is also involved in promoting NPC metastasis [137]. Li and coworkers [138] showed that both LMP1 and miR-10b are over expressed in metastatic EBV+ human NPC C666-1 cell. The levels of miR-10b were significantly reduced in LMP1-silent C666/shLMP cells, while dramatically elevated in LMP1-over-expressing C666/LMP cells, which indicated that LMP1 expression was positively associated with miR-10b expression in NPC cells. Interestingly, inhibition of Twist expression by siRNA dramatically reduced miR-10b levels in LMP1 expressing cells, but not in the LMP1-silent C666/shLMP and miR-10b over expressing C666/shLMP/miR-10b cells. The authors proposed that LMP1 may induce the expression of Twist, which in turn up-regulates miR-10b transcription in NPC cells. Induction of miR-10b overexpression in LMP1-silent C666-1 cells promoted significant wound healing and transmembrane invasiveness *in vitro*. More importantly, miR-10b over-expression promoted the metastasis of NPC and accelerated the death of tumor-bearing nude mice.

A recent report [139] showed that metastatic human HNSCC are associated with low levels of *TAp63*, a *p53* family member that acts a tumor suppressor gene, [140]. Metastatic HNSCC also exhibited reduced levels of Dicer, the endoribonuclease involved in miRNA generation that has been shown to function as a haploinsufficient tumor suppressor [141]. *TAp63* binds to and transactivates the Dicer promoter, suggesting a role in metastasis through alteration of miRNA processing. Indeed, miR-130b [142] was down-regulated in metastatic HNSCCs cells lacking *TAp63*. Based on these data, the authors concluded that the participation of *TAp63* in tumor and metastasis suppression involves the coordinate transcriptional regulation of Dicer and miR-130b [139].

Computational genome-scale predictive strategies were also used to predict down-regulated miRNAs in HNSCC [143]. The expression of one of these miRNA, miR-204, was highly reduced in HNSCC tumors and this was related to the genomic imbalanced 9q21.1–22.3 locus corresponding to host gene transient receptor potential melastatin 3 cation channel (TRPM3) that has been associated with genetic predisposition for head and neck cancer [144]. Predicted miR-204 targets that are up-regulated in HNSCC are significantly related through their biological functions, mainly participating in proteolysis, cell adhesion, cell cycle regulation and cell proliferation. Expression profiling and computational biological approaches confirmed that miR-204 suppression could up-regulate genes involved in cell cycle and extracellular matrix remodeling associated to cancer metastasis and/or poor prognosis. Indeed, miR-204 overexpression is sufficient to suppress cell-matrix interaction, motility and invasiveness *in vitro*, as well as experimental lung colonization of SQ38 HNSCC tumors *in vivo*, demonstrating that it represents a potent suppressor of metastasis. Expression pattern of 19 miR-204 targets identified a subtype of HNSCC tumors exhibiting an EGFR-pathway signature [145] and predicted earlier relapse, which suggested a potentially important role of miR-204 in HNSCC prognosis. The discovery of molecular networks regulated by microRNAs could be exploited for the design of new treatments as an alternative to the single-gene target paradigm.

### 5.7. MetastamiRs in Cervical Carcinoma

Cervical carcinoma (CC) is a leading cause of death among women worldwide, with an estimated incidence of 470,000 new cases and 233,000 deaths per year [146]. Epidemiologic and experimental studies have identified a causal role of high risk HPV types in cervical carcinogenesis, mainly HPV 16 and HPV 18 [147]. Persistent viral infection in combination with strong, constitutive expression of E6 and E7 viral oncogenes is a necessary step for malignant transformation because these proteins interact with p53 and pRB proteins leading to their degradation and deregulation of the cell cycle [148,149]. Moreover, E6 and E7 target additional cellular proteins and transcriptional regulators [150,151] including c-MYC [152], and Skip [153]. Finally, at DNA level, E6 and E7 deregulate cell proliferation and induce genetic instability, which promote the accumulation of mutations and aneuploidy [154]. HPV oncoproteins play an important role in miRNA expression by means of p53 degradation induced by E6 and E7, which induces pRB degradation and subsequently, E2F release from the pRB-e2F complex, which leads to overexpression of the transcription factor c-MYC. c-MYC induces the expression of the miR-17-92 family [155], a miRNA polycistron also known as oncomir-1 that is upregulated in a wide variety of human tumors [156,157]. p53 regulates cellular miRNAs by transcriptional activation of miR-23a, -26a and 3-4a. On the other hand, p53 decreases expression of distinct miRNA clusters, such as miR-106b/miR-93/miR-25, miR-17-15p/18a/19a/20a/19b-1/92-1 and miR-106a/18b/20b/19b-2, through an indirect mechanism which involves repression of E2F1 [158]. As it can be noted, E6 and E7 viral oncoproteins exert an important role on cervical epithelia miRNA expression profiles.

A few reports have explored the role of potential metastatic or anti-metastatic miRNAs in CC. In CC cells, Qiang and colleagues evidenced a marked overexpression of Plexin B1, the receptor for Sema4D that has been reported to trigger multiple cellular responses, including metastasis in various types of tumor cells. The high Plexin B1 overexpression inversely correlated with miR-214 levels in normal and tumoral samples, which suggested that miR-214 could have distinct roles during the inhibition of malignant phenotype, including cell cycle induction, migration and metastasis, probably mediated by Plexin B1 inhibition [159].

Using microarray expression profile to analyze 96 cancer-related miRNAs, Hu and coworkers were able to identify ten miRNAs (miR-200a, miR-9, miR-10b, miR-183, miR-204, miR-24, miR-181a, miR-193b, miR-146b and miR-10a) related to cervical cancer survival. Notably, functional characterization indicated that miR-200a regulates cell adhesion and cancer cell metastasis. Hu and colleagues proposed that miR-200a expression inhibition during CC carcinogenesis process could lead to increased cancer cell motility. Possible targets for miR-200a are ZEB1/2, TGFB2 AND EXOC5, which are related to the metastatic potential of tumor cells [160]. Finally, Lee and collaborators showed that there is a miRNA deregulation in early stage of cervical cancer progression, in comparison with normal cervical epithelial tissues; moreover, miR-127 could regulate lymph node metastasis [161].

### 5.8. MetastamiRs in Hepatocellular Carcinoma

Primary liver cancer mainly refers to hepatocellular carcinoma (HCC), cholangiocarcinoma, and hepatic angiosarcoma. HCC is considered as the third leading cause of death from cancer, primarily due to late symptom manifestation and unresponsiveness to treatment [162]. Hence, patients affected with HCC have a 5-year survival rate [163]. Persistent hepatitis B virus (HBV) infection is a leading cause to develop HCC. Molecular evidence suggested that viral X-gene product (HBx) has the major role in the etiology of HCC [164]. HBx produces a 154 amino acids protein that has been implicated as a cofactor or tumor promoter through its pleiotropic regulatory functions in apoptosis, DNA repair, chromosomic instability, proliferation, inflammation and tumorigenesis [165], through the activation of transcription factors such as NF-κB, c-MYC and TNF-α [166,167]. Therefore, this viral pleiotropic protein could have an important indirect effect on miRNA expression profile through the activation of key transcription factors.

In normal liver, miR-122 corresponding to about 70% of total miRNA population is considered as a liver specific miRNA implicated in systemic metabolism. During HCC carcinogenesis, miR-122 expression is lost; moreover restoration of miR-122 expression in cell lines can suppress malignant growth by directly targeting cyclin G1 expression and metastatic properties [168–170]. miR-195, one of the miR-16/15/195/424/497 family members, is down-regulated in a wide variety of tumors including colorectal cancer; moreover, it seems to negatively regulate lymph-node metastasis by targeting cyclins *D1* and *E1*, *CDK6*, *E2F3*, suppressing tumorigenesis and blocking G1-S transition. miR195 down-regulation during HCC process suggested that miR-195 could have an important role in the control of processes that are deregulated in HCC carcinogenesis [171].

miR-21 contributes to HCC growth and maintenance mechanisms, probably through AKT and ERK pathway activation. It promotes angiogenesis by means of HIF-1a stabilization. miR-21 is also involved in HCC metastasis and migration by directly targeting *PTEN*. This metastamiR can activate specific matrix metalloproteinases, such as MMP2 and MMP9, that are downstream mediators of *PTEN* [172]. *PTEN* interacts with the focal adhesion kinase FAK and reduces its tyrosine phosphorylation. Therefore, *PTEN* is an antagonist of metastasis by negatively regulating cell interactions with the extracellular matrix. Other microRNAs, such as miR-221 and miR-222, are able to deregulate *PTEN*. Moreover, these miRNAs also target the protein phosphatase 2A subunit B (PPP2R2A) and TIMP3, thus activating the AKT pathway and metalloproteases associated to cell invasion and metastasis [84,173].

TGF-beta is a key regulator of metastasis, which can regulate hallmarks of cancer that finally lead to metastasis, such as cell proliferation, differentiation, motility, adhesion and programmed cell death. Recently, Wang and co-workers have shown that miR-181b is transcriptionally induced by TGF-beta signaling pathway through SMAD4. Moreover, miR-181 targets TIMP3 that is an inhibitor of metalloprotease, inductor of apoptosis and inhibits angiogenesis, cell migration and invasion [174]. It is clear that the induction of metastasis through TGF-beta/SMAD4/miR-181b leads to inhibition of metastatic negative regulators such as TIMP3 and over-activation of metalloproteinases. Two additional microRNA that are associated to HCC metastasis are miR-143 whose expression is mediated by NF-κB [175] and miR-17-5p whose activity depends on p38 mitogen-activated protein kinase and increased phosphorylation of heat shock protein 27 [176].

## 6. Conclusions

**Figure 3:**
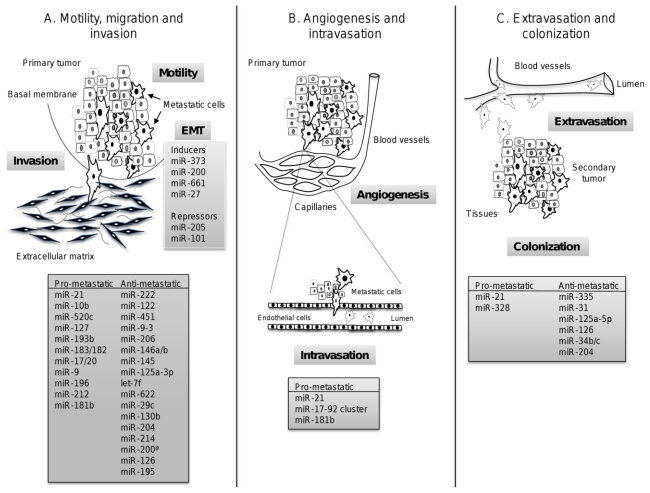
MicroRNAs implicated in the regulation of metastasis. miRNAs are grouped according their roles in metastatic processes: (**A**) Invasion, motility and migration; (**B**) angiogenesis and intravasation; and (**C**) extravasation and colonization of the secondary organ. Characterized metastamiRs that conferred (pro-metastatic) or repressed (anti-metastatic) metastatic abilities to tumoral cells are denoted in gray boxes.

The recent discovery of the role of microRNAs as tumor-suppressor genes and oncogenes has added an additional level of complexity to the mechanisms leading to tumorigenesis. In particular, the extensive review presented here evidences that metastamiRs have emerged as new molecular players to regulate invasion and metastasis events in all types of cancers, including lung, prostate, colorectal, gastric, head and neck, cervical and hepatocellular carcinomas. Some of these miRNAs are common regulators of cell motility and invasion in distinct cancers (Figure 3); others appear to be cancer specific, according to the current knowledge. Understanding how metastamiRs are involved in regulating tumor invasion and metastasis process will provide a promising strategy for the identification of molecular markers for progression and prognosis, for response to chemotherapy, early biomarkers of aggressive tumors, and the development of new metastamiRs-based treatments. However, further investigations about the role of each miRNA in each cancer are required in order to use them as targets for therapy, prognosis and diagnosis in the near future.

## Abbreviations

miRNA: microRNA
RNA pol II: RNA polymerase II
DGCR8: DiGeorge syndrome critical region gene 8
pri-miRNA: primary-miRNA
pre-miRNA: precursor miRNA
pre-mRNA: precursor mRNA
3′-UTR: 3′ untranslated region
RISC: RNA-induced silencing complex
TRBP: TAR RNA-binding protein
AGO: Argonaute
